# Subjective symptoms are triggers for the detection of immune checkpoint inhibitor-induced interstitial lung disease and associate with disease severity: a single-center retrospective study

**DOI:** 10.1186/s40780-024-00373-7

**Published:** 2024-08-27

**Authors:** Mari Yokoi, Atsushi Yonezawa, Daiki Hira, Tomohiro Handa, Kiminobu Tanizawa, Shunsaku Nakagawa, Masahiro Tsuda, Yasuaki Ikemi, Ryo Itotani, Hironori Yoshida, Motoo Nomura, Junichi Matsubara, Kosaku Murakami, Hiroaki Ozasa, Manabu Muto, Tomohiro Terada

**Affiliations:** 1 Department of Clinical Pharmacology and Therapeutics, Kyoto University Hospital, 54 Shogoin Kawahara-cho, Sakyo-ku, Kyoto, 606-8507 Japan; 2 Division of Integrative Clinical Pharmacology, Faculty of Pharmacy, Keio University, 1-5-30 Shibakoen, Minato-ku, Tokyo, 105-8512 Japan; 3 Department of Advanced Medicine for Respiratory Failure, Graduate School of Medicine, Kyoto University, 54 Shogoin Kawahara-cho, Sakyo-ku, Kyoto, 606-8507 Japan; 4 Department of Respiratory Medicine, Graduate School of Medicine, Kyoto University, 54 Shogoin Kawahara-cho, Sakyo-ku, Kyoto, 606-8507 Japan; 5 Graduate School of Pharmaceutical Sciences, Kyoto University, 46-29 Yoshida Shimo-Adachi-cho, Sakyo-ku, Kyoto, 606-8501 Japan; 6 Department of Clinical Oncology, Kyoto University Hospital, 54 Shogoin Kawahara-cho, Sakyo-ku, Kyoto, 606-8507 Japan; 7 Department of Head and Neck Oncology and Innovative Treatment, Graduate School of Medicine, Kyoto University, 54 Shogoin Kawahara-cho, Sakyo-ku, Kyoto, 606-8507 Japan; 8 Department of Medical Oncology, Graduate School of Medicine, Kyoto University, 54 Shogoin Kawahara-cho, Sakyo-ku, Kyoto, 606-8507 Japan; 9 Division of Clinical Immunology and Cancer Immunotherapy, Center for Cancer Immunotherapy and Immunobiology, Graduate School of Medicine, Kyoto University, 53 Shogoin Kawahara-cho, Sakyo-ku, Kyoto, 606-8507 Japan

**Keywords:** Immune checkpoint inhibitors, Immune-related adverse events, Drug-induced interstitial lung disease, Subjective symptoms

## Abstract

**Background:**

Interstitial lung disease (ILD) is one of the most common fatal immune-related adverse events (irAEs). ILD development adversely affects the continuation of anticancer drug therapy, including immune checkpoint inhibitor (ICI) therapy and prognosis. There are no established useful clinical indicators for the early detection of ILD. Furthermore, the factors that lead the attending physician to suspect ICI-induced ILD (ICI-ILD) remain unclear. This study aimed to investigate the ICI-ILD detection based on subjective symptoms and their relationship with disease severity in patients receiving anti-PD-1/PD-L1 antibody.

**Methods:**

This was a retrospective observational study. We enrolled the patients who received anti-PD-1/PD-L1 antibody at Kyoto University Hospital between September 2014 and April 2021. Patients who developed ICI-ILD were stratified into two distinct groups based on factors that triggered the suspicion of ILD development. The “Subjective symptoms” group was defined as patients in whom ILD was detected based on subjective symptoms. Conversely, the “Routine examinations” group was defined as patients in whom ILD was suspected based on scheduled routine examinations. The severity of ILD in each group was assessed and its association with changes in the respiratory symptoms was examined.

**Results:**

Of 926 patients who received anti-PD-1/PD-L1 antibody, 51 patients (5.5%) developed ICI-ILD. The incidence of ICI-ILD in patients with lung cancer was significantly higher than that in patients with other cancers (*P* < 0.001). Among the patients with ICI-ILD, 27 patients (52.9%) were classified into the “Subjective symptoms” group. The “Subjective symptoms” group exhibited a significantly higher proportion of Grade 3–5 ICI-ILD cases than the “Routine examinations” group (76.2% vs. 23.8%, *P* = 0.010). At the last visit, before the suspected onset of ILD, 21 of the 27 patients (77.8%) had no symptoms or no change in the respiratory symptoms.

**Conclusion:**

Subjective symptoms triggered the suspicion of Grade 3–5 ICI-ILD. Enhanced monitoring and patient education could be essential for the early detection of ICI-ILD because ILD may develop rapidly. Our findings might help to manage ICI-ILD in clinical practice.

## Background

Interstitial lung disease (ILD) is one of the most common fatal immune-related adverse events (irAE). Anti–PD-1/PD-L1 antibody monotherapy causes a wide range of irAEs, including ILD (35%), hepatitis (22%), colitis (17%), neurological events (15%), and myocarditis (8%), which are often fatal when the vital organs are involved [1]. The development of ILD adversely affects the continuation of anticancer drug therapy, including immune checkpoint inhibitors (ICI), and patients’ prognosis. A Japanese multicenter study on patients with non-small cell lung cancer (NSCLC) treated with nivolumab reported that 10.4% of patients developed ILD, and 41.5% of them developed ILD of grade 3 or higher [2]. As for the poor prognostic factors for ICI-induced ILD (ICI-ILD), a study on nivolumab in NSCLC identified a diffuse alveolar damage-like radiographic pattern, onset of ILD ≤ 60 days from the initial dose of nivolumab, pleural effusion present before nivolumab treatment, abnormal opacities distributed contralateral to the tumor or bilaterally, and abnormal changes in C-reactive protein (CRP) levels [3]. In another study on ICIs for NSCLC, the predictive factors of severe irAEs were age ≥ 65 years, percent change in derived neutrophil-to-lymphocyte ratio (dNLR) before and after ICI treatment ≥ 0.2, percent change in lymphocyte-to-monocyte ratio (LMR) before and after ICI treatment < -0.25, and a post-advanced lung cancer inflammation index ≥ 17 [4]. Severe irAEs were resolved with immunomodulatory agents earlier than mild-to-moderate irAEs [5]. This suggests that early detection and treatment of ICI-ILD is important for its outcome. However, there are no established useful clinical indicators for the early detection of ILD.

The onset timing of irAEs resulting from the administration of ICIs exhibits a wide range, and a long-term multidisciplinary follow-up should be arranged [5]. Because late-onset dose-limiting toxicity is a challenge in ICI treatment, monitoring for immune-related toxicity for an adequate period of time is clinically important [6]. In clinical practice, ILD is suspected based on laboratory tests, saturation of percutaneous oxygen (SpO_2_), and subjective symptoms (such as dry cough and dyspnea), and high-resolution computed tomography (HRCT) is usually used to diagnose ILD. ICI therapy is primarily administered on an outpatient basis and the administration interval extends to a maximum of six weeks. Therefore, subjective symptoms that the patient can assess are considered important factors in suspecting ILD development. A previous report showed that subjective symptom questionnaires were useful in the detection of ICI-ILD [7]. However, the frequency of subjective symptoms triggering ILD diagnosis and the relationship between subjective symptoms and disease severity remain unclear.

In this study, we investigated the suspicion of ICI-ILD based on subjective symptoms and their relationship with ICI-ILD severity in patients receiving ICI.

## Methods

### Subjects and study design

This was a retrospective observational study. Patients who received one of the following five anti-PD-1/PD-L1 antibodies (nivolumab, pembrolizumab, atezolizumab, durvalumab, or avelumab) at Kyoto University Hospital between September 2014 and April 2021 were enrolled.

Patients suspected of having ICI-ILD were identified from electronic medical records (EMR) database. One pulmonologist retrospectively reassessed the grade of ILD at diagnosis according to “pneumonitis” in the Common Terminology Criteria for Adverse Events (CTCAE) v5.0. In the case of patients who died from ILD, however, the grade was defined as Grade 5.

### Investigations

The onset of ILD was identified using HRCT imaging data. The demographic data (age at the start of anti-PD-1/PD-L1 antibody, sex, and cancer type), drug data (anti-PD-1/PD-L1 antibody and combinations), and ICI-ILD, and laboratory data (respiratory symptoms, CRP, NLR, dNLR, and LMR at the onset of ICI-ILD) were extracted from the EMR database. The laboratory data closest to the HRCT imaging data used for the diagnosis of ICI-ILD were utilized. Data that exceeded a range of ± 6 days from the date of HRCT image diagnosis were excluded.

Patients receiving anti-PD-1/PD-L1 antibody on an outpatient basis completed a questionnaire regarding the subjective symptoms on each day of administration. It was recorded in the EMR database by the medical staff. The subjective symptoms were collected from the records based on the results of a questionnaire and interviews with physicians, pharmacists, and nurses. The patients who developed ICI-ILD were stratified into two distinct groups based on factors that triggered the suspicion of ILD development. The “Subjective symptoms” group was defined as patients in whom ILD was suspected based on subjective symptoms. Conversely, the “Routine examinations” group was defined as patients in whom ILD was suspected based on scheduled routine examinations, such as SpO_2_, complete blood count, CRP, chest radiographs, and HRCT for treatment effect. Basically, SpO_2_ levels and blood tests were conducted when the patients visited the hospital with each administration of chemotherapy. HRCT was performed every 2–3 months for tumor evaluation. In some cases, those tests were conducted more frequently depending on other department visits and the patient’s condition. Patients who complained of subjective symptoms during routine visits and were suspected of ILD development were defined as the “Subjective symptoms” group. On scheduled visits or during hospitalization, the physician might suspect ICI-ILD based on HRCT, SpO_2_ or blood test results before ascertaining the presence of subjective symptoms. In such cases, since the initial suspicion of ILD is based on tests, these instances were classified under “Routine examinations” group. The reason for this grouping was to investigate the frequency of suspecting the development of ILD by subjective symptom complaints.

The onset or worsening of subjective symptoms could be evaluated from the records of the medical staff’s interviews, and questionnaires in the EMR database. The date of the visit when ILD was diagnosed was designated as “Visit 0”. Starting from “Visit 0”, the previous visit dates were defined sequentially as “Visit − 1” and “Visit − 2”. Changes in the subjective symptoms from Visit − 2 to Visit − 1 were categorized as “no symptoms”, “no change”, “worsening symptoms”, or “new symptoms”.

### Matched case-control study

The case patients included all individuals diagnosed with ICI-ILD in the retrospective observational study. Control patients without ICI-ILD (non-ICI-ILD patients) were matched with patients who developed ICI-ILD. For each ICI-ILD patient, one non-ICI-ILD patient was randomly selected, with matching for age (± 10 years), sex, cancer type, anticancer drug regimen, and duration of anti-PD-1/PD-L1 Ab administration (longer than that of the each ICI-ILD patient) which were reported as risk factors for the onset and severity of ICI-ILD [4, 8–10]. Regarding the presence of subjective symptoms in the case patients, they were considered symptomatic if they experienced the new onset or worsening of respiratory symptoms (dyspnea, cough, or fever) at the time of ICI-ILD diagnosis. The control patients were considered symptomatic if they experienced the new onset or worsening of respiratory symptoms (dyspnea, cough, or fever) within a period equivalent to the duration from the first administration of anti-PD-1/PD-L1 Ab to the ICI-ILD diagnosis in the matched case patients. The results were presented as odds ratios (ORs) with 95% confidence intervals (CIs).

### Statistical analysis

The chi square test was used to determine the incidence of ICI-ILD, and the Fisher’s exact test was used to determine the severity of ICI-ILD. Fisher’s exact test was used to determine the relationship between subjective symptoms and ICI-ILD detection or ICI-ILD severity. Bonferroni-Corrected test was used as a multiple comparison test for subjective symptoms. Statistical significance was defined as a two-sided *P* value of < 0.05, and all data analyses were performed using GraphPad Prism version 9.5.1 (GraphPad Software Inc., San Diego, USA).

## Results

### Patient characteristics

Of the 926 patients who received anti-PD-1/PD-L1 antibody, 51 patients (5.5%) developed ICI-ILD. As shown in Table 1, nivolumab was the most used anti-PD-1/PD-L1 antibody, followed by pembrolizumab, atezolizumab, durvalumab, and avelumab. A total of 205 patients (22.1%) received anti-PD-1/PD-L1 antibody plus ipilimumab or combination chemotherapy. Among the patients who received anti-PD-1/PD-L1 antibody, 458 patients (49.5%) had lung cancer.

**Table 1:** Clinical features of 926 patients who received anti-PD-1/PD-L1 antibody

		Total (*n* = 926)
Age, n (%)	≥ 65	641 (69.2)
	< 65	285 (30.8)
Sex, n (%)	Male	624 (67.4)
	Female	302 (32.6)
Anti-PD-1/PD-L1 antibody, n (%)	Nivolumab	526 (56.8)
	Pembrolizumab	234 (25.3)
	Atezolizumab	213 (23.0)
	Durvalumab	39 (4.2)
	Avelumab	4 (0.4)
Combination, n (%)	Ipilimumab	40 (4.3)
	Chemotherapy	165 (17.8)
Cancer type, n (%)	Lung cancer	458 (49.5)
	Malignant melanoma	101 (10.9)
	Gastric cancer	68 (7.3)
	Bladder cancer	57 (6.2)
	Kidney cancer	55 (5.9)
	Head and neck cancer	40 (4.3)
	Esophageal cancer	39 (4.2)
	Other	108 (11.7)

### Incidence and severity of ICI-ILD

Overall, 51 patients developed ICI-ILDs (5.5%), 37 patients with lung cancer, and 14 patients were with other cancers. The incidence of ICI-ILD in patients with lung cancer was significantly higher than that in patients with other cancers (8.1% vs. 3.3%, *P* < 0.001).

Regarding the severity of ICI-ILDs, Grade 3–5 ILD was observed in 21 patients (41.2%) and Grade 5 ILD in seven patients (13.7%). As shown in Table 2, the percentage of patients with Grade 3–5 ILD was not significantly different between those with lung cancer and those with other cancers (43.2% vs. 35.7%, *P* = 0.754).

**Table 2:** Severity of ICI-ILD

	Grade 1–2 (*n* = 30)	Grade 3–5 (*n* = 21)	*P* value
Lung cancer patients	21 (56.8)	16 (43.2)	0.754
Other cancer patients	9 (64.3)	5 (35.7)	

Fisher’s exact test was used. ICI-ILD, immune checkpoint inhibitor -induced interstitial lung disease; ILD, interstitial lung disease

### Clinical characteristics of the ICI-ILD patients

A comparison of the clinical characteristics according to the ILD severity was shown in Table 3. The patient age was at the time of the first anti-PD-1/PD-L1 antibody administration, and laboratory tests were performed at the time of ICI-ILD onset. CRP level, NLR, and dNLR at the onset of ICI-ILD were significantly higher in patients with Grade 3–5 ILD than in those with Grade 1–2 ILD (*P* = 0.004, 0.002, 0.002). On the other hand, age and anti-PD-1/PD-L1 antibody duration until ILD onset were not significantly different (*P* = 0.196, 0.147).

**Table 3:** Comparison of the clinical characteristics according to the ILD Severity

		Grade1-2 (*n* = 30)	Grade3-5 (*n* = 21)	*P* value
Age, n	≥ 65	25	14	0.196
	< 65	5	7	
Sex, n	Male	28	19	1.000
	Female	2	2	
Anti-PD-1/PD-L1 antibody, n	Nivolumab	19	9	0.148
	Pembrolizumab	7	8	0.351
	Atezolizumab	1	3	0.293
	Durvalumab	3	1	0.635
Combination, n	Chemotherapy	2	2	1.000
Anti-PD-1/PD-L1 antibody duration until ILD onset (cycles), median (range)		4.5 (1–71)	3.0 (1–13)	0.147
Cancer type, n	Lung cancer	21	16	0.754
	Other cancer	9	5	
	Malignant melanoma	3	1	-
	Gastric cancer	1	0	-
	Bladder cancer	0	3	-
	Kidney cancer	1	0	-
	Head and neck cancer	2	1	-
	Esophageal cancer	2	0	-
Laboratory test, median (range)	Onset CRP	2.50 (0.10–22.00)	8.50 (1.10–23.10)	0.004
	Onset NLR	5.45 (1.21–24.57)	11.09 (3.35-99.00)	0.002
	Onset dNLR	2.91 (0.73–9.99)	5.07 (2.03-99.00)	0.002
	Onset LMR	21.63 (0.40–5.56)	1.36 (0.25-7.00)	0.276

Fisher’s exact test and the Mann-Whitney U test were used. ICI-ILD, immune checkpoint inhibitor -induced interstitial lung disease; ILD, interstitial lung disease; CRP, C-reactive protein; NLR, neutrophil-to-lymphocyte ratio; dNLR, derived NLR; LMR, lymphocyte-to-monocyte ratio

### Frequency of subjective symptoms as a diagnostic trigger for ICI-ILD and its association with ILD severity

First, we carried out a matched case-control study with a control group of patients without ICI-ILD to examine the relationship between subjective symptoms and ILD development. The new onset or worsening of subjective symptoms tended to, but not significantly, increase the risk of all grades of ICI-ILD (OR: 2.21, 95% CI: 1.00-4.73, Table 4). Therefore, we examined the findings that led to the suspicion of ILD in patients with each grade of ICI-ILD. Among the 51 patients who developed ICI-ILD, 36 patients (70.6%) used a questionnaire about subjective symptoms, and all patients had medical records regarding subjective symptoms, regardless of the type of visit. Among the patients who developed ICI-ILD, 27 patients (52.9%) were in the “Subjective symptoms” group. As shown in Table 5, the “Subjective symptoms” group exhibited a significantly higher proportion of Grade 3–5 ICI-ILD cases than the “Routine examinations” group (*P* = 0.010). Among grade 5 ILD patients, six of seven patients were in the “Subjective symptoms” group. The “Subjective symptoms” group exhibited a higher proportion of grade 5 ICI-ILD cases than the “Routine examinations” group (22.2% vs. 4.2%, *P* = 0.103).

Further analysis was performed in patients suspected of developing ICI-ILD based on subjective symptoms. We evaluated the changes in the subjective symptoms. The median date of Visit − 1 was 14 days (range: 4–26 days) before Visit 0. On the date of Visit − 1, 21 patients (77.8%) had no symptoms or no change in respiratory symptoms, and six patients (22.2%) had new or worsening symptoms.

**Table 4:** Relationship between subjective symptoms and the detection of ILD

		Non-ICI-ILD (*n* = 51)	ICI-ILD (*n* = 51)	OR	95% CI	*P* value
Subjective symptoms, n	Yes	20	30	2.21	1.00-4.73	0.07
	No	31	21			

Fisher’s exact test was used. ICI-ILD, immune checkpoint inhibitor-induced interstitial lung disease; ILD, interstitial lung disease; CI, confidence interval; OR, odds ratio

### Association of individual subjective symptoms with ICI-ILD severity

The proportion of patients with subjective symptoms according to the severity of ICI-ILD is shown in Table 5. The incidence of dyspnea was markedly higher in patients with Grade 3–5 ILD than in those with mild ILD (*P* < 0.001). All patients with Grade 5 ILD patients had dyspnea. In lung cancer patients, dyspnea was observed in three out of 21 cases (14.3%) with Grade 1–2 ILD compared to 11 out of 16 cases (68.8%) with Grade 3–5 ILD, with a significance of *P* = 0.002. In other cancer patients, dyspnea was observed one out of nine cases with Grade 1–2 ILD compared to five out of five cases with Grade 3–5 ILD, with a significance of *P* = 0.003.

**Table 5:** Relationship between subjective symptoms and the severity of ILD

		Grade 1–2 (*n* = 30)	Grade 3–5 (*n* = 21)	*P* value^a^
Factors in the Detection of ICI-ILD, n (%)	Subjective symptoms	11 (36.7) [G1:0, G2:11]	16 (76.2) [G3:6, G4:4, G5:6]	0.010
	Routine examinations	19 (63.3) [G1:10, G2:9]	5 (23.8) [G3:2, G4:2, G5:1]	
Subjective symptoms, n (%)	Dyspnea	4 (13.3) [G1:0, G2:4]	16 (76.2) [G3:5, G4:4, G5:7]	< 0.001
	Cough	8 (26.7) [G1:0, G2:8]	7 (33.3) [G3:5, G4:0, G5:2]	0.757
	Fever	6 (20.0) [G1:0, G2:6]	6 (28.6) [G3:3, G4:2, G5:1]	0.518

Fisher’s exact test was used. ^a^ Bonferroni-Corrected test was used as a multiple comparison test for subjective symptoms. Statistical significance was defined as a two-sided *P* value of < 0.017. ICI-ILD, immune checkpoint inhibitor-induced interstitial lung disease; ILD, interstitial lung disease

## Discussion

ILD is a fatal irAE that causes subjective symptoms such as dry cough and dyspnea [1, 8]. Although the first criterion in the decision tree for ICI-ILD treatment is the presence of new respiratory symptoms during receiving ICI [9], it is unclear what factors lead physicians to suspect ICI-ILD. In this study, we investigated the factors that may lead to the suspicion of ICI-ILD in patients receiving anti-PD-1/PD-L1 antibody, and showed that the new onset or worsening of subjective symptoms tended to, but not significantly, increase the risk of all grades of ICI-ILD. In addition, we examined the relationship between subjective symptoms and the ICI-ILD severity. Among the patients diagnosed with ICI-ILD, more than half of them were suspected based on subjective symptoms. In particular, Grade 3–5 or fatal ICI-ILD was more frequently detected based on subjective symptoms than Grade 1–2 ICI-ILD. Shortness of breath and dyspnea on the questionnaire were reported to be useful in detecting the onset of ICI-ILD [7]. Our results suggest that ILDs that are diagnosed based on dyspnea are more likely to detect Grade 3–5 ICI-ILD, and that it is important to diagnose ILDs at an earlier stage.

Interestingly, most patients had no respiratory symptoms or no change in the respiratory symptoms on the date of the most recent visit from the date of the visit that triggered the diagnosis of ILD. Anti-PD-1/PD-L1 antibodies are usually administered once every 2–4 weeks. Most patients visited the hospital for the administration of medicines. According to our results, in patients with Grade 3–5 ICI-ILD requiring hospitalization, the median number of days from respiratory symptom onset to the hospital visit was four days (0–14 days). Furthermore, 80% of patients have unscheduled visits triggered by reports made by individuals other than the patients [10]. In addition, the onset timing of irAEs has a wide range [5]. Therefore, the early detection of rapidly developing ICI-ILD is not easy. Glucocorticoid is the standard therapy for irAE management. Only patients treated with methylprednisolone pulse therapy (primarily for Grade 3–5 ILD and hepatitis) had shorter progression-free survival and overall survival, and patients treated with low-to-high doses of glucocorticoids had equivalent survival to those who did not receive glucocorticoid [11]. The early detection and initiation of glucocorticoid therapy may obviate pulse therapy, which is associated with a poor prognosis. Recently, subcutaneous injection preparations of ICI have been being developed [12], which may increase the interval between regular outpatient visits and may lead to delay in ILD detection. Recently, a web application was developed that allows patients to check for irAE [13]. Patients should be provided with information such as symptom-specific response flowcharts and web applications so that they can identify which symptoms are emergencies at any time. It is important for outpatients to self-monitor their symptoms and contact a medical facility when symptoms appear.

Subjective symptoms are useful in suspecting the development of ICI-ILD. However, it is important to predict ILD before its onset or detect it at a milder stage. As for poor prognostic factors for ICI-ILD, abnormal changes in CRP levels, percent change in dNLR before and after ICI treatment ≥ 0.2, and percent change in LMR before and after ICI treatment < -0.25 have been reported [3, 4]. Although the prognostic markers for ICI-ILD have been identified, no markers have been confirmed to be useful for the early detection of ICI-ILD onset. Therefore, it is necessary to investigate the objective indicators that can predict the development of ICI-ILD and studies are required in the future.

This study has several limitations. First, this was a retrospective study using EMR database. Subjective symptoms were not recorded unless the patients complained of them, and they were not always recorded in the EMR database. However, physicians routinely took a caution about the subjective symptoms caused by anticancer drugs and described them in EMR database. A medical questionnaire common to ICI was not always checked every visit and might not be recorded, so missing data was inevitable. Therefore, it will be necessary to collect the data prospectively or use a web application in the future. Second, we could not investigate the subjective symptoms in patients who did not develop ICI-ILD. Subjective symptoms can be used in the differential diagnosis of other respiratory diseases. Therefore, the proportion of patients with ICI-ILD among those who had subjective symptoms remains unclear. Finally, the proportion of patients receiving combination therapy was small because the regimens approved in Japan after May 2021 were not included. The incidence and severity of ICI-ILDs are expected to increase further with the current approval of many combination therapies. Therefore, it is necessary to continue studying and revise these responses.

## Conclusions

Subjective symptoms were the trigger for the suspicion of Grade 3–5 ICI-ILD. Because ILD may develop rapidly, enhanced monitoring of subjective symptoms and patient education are important for the early detection of ICI-ILD. Our findings can help manage ICI-ILDs in clinical practice.

## Data Availability

The datasets used and/or analyzed is the present study are available from the corresponding author upon reasonable request.

## Abbreviations

CI: Confidence interval
CRP: C-reactive protein
CTCAE: The Common Terminology Criteria for Adverse Events
dNLR: Derived neutrophil-to-lymphocyte ratio
EMR: Electronic medical record, HRCT, high-resolution computed tomography
ICI: Immune checkpoint inhibitor
ICI-ILD: Immune checkpoint inhibitor-induced interstitial lung disease
ILD: Interstitial lung disease
irAE: Immune-related adverse event
LMR: Lymphocyte-to-monocyte ratio
NSCLC: Non-small cell lung cancer
OR: Odds ratio
SpO_2_: Saturation of percutaneous oxygen
